# Single-Port Laparoscopy Compared with Conventional Laparoscopic Surgery: A Systematic Review and Meta-Analysis

**DOI:** 10.3390/jcm14144915

**Published:** 2025-07-11

**Authors:** Baudolino Mussa, Barbara Defrancisco, Ludovico Campi, Mario Morino

**Affiliations:** 1 Surgical Science Department, University of Turin, 10124 Turin, Italy; ludovico.campi@edu.unito.it (L.C.); mario.morino@unito.it (M.M.); 2 Alchemica Torino, Via San Marino 25, 10136 Turin, Italy; barbyd77@gmail.com

**Keywords:** single-port laparoscopy, minimally invasive surgery, meta-analysis, systematic review, surgical outcomes, patient satisfaction

## Abstract

**Background/Objectives**: Single-port laparoscopy represents a significant advancement in minimally invasive surgical techniques and is designed to reduce surgical trauma and enhance cosmetic outcomes. However, ongoing debate surrounds its relative benefits and limitations as compared with conventional multi-port laparoscopy. This study systematically reviewed and analyzed comparative outcomes between these two approaches. **Methods**: We conducted a comprehensive systematic search of major electronic databases from January 2000 to October 2023, following PRISMA guidelines. Only randomized controlled trials comparing single-port laparoscopy with conventional laparoscopy were included. We analyzed operative outcomes, postoperative recovery parameters, complications, and patient-reported measures using random-effects models, with heterogeneity explored through subgroup analyses. **Results**: Forty-three randomized controlled trials involving 5807 patients were analyzed. Single-port laparoscopy demonstrated longer operative times (weighted mean difference: +10.5 min; 95% CI: 7.83–13.18; *p* < 0.001), superior cosmetic satisfaction (standardized mean difference: +0.61; 95% CI: 0.39–0.83; *p* < 0.001), and reduced postoperative pain within 24 h (standardized mean difference: −0.58; 95% CI: −0.95 to −0.21; *p* = 0.002). The overall complication rates showed no significant differences (risk ratio: 0.94; 95% CI: 0.78–1.14; *p* = 0.31), though incisional hernia risk increased with single-port laparoscopy (odds ratio: 2.26; 95% CI: 1.23–4.15; *p* = 0.009). **Conclusions**: Single-port laparoscopy offers meaningful improvements in cosmetic outcomes and early pain relief, balanced against longer operative times and increased hernia risk. The substantial heterogeneity observed underscores the importance of surgeon experience, appropriate patient selection, and optimal technique selection in determining outcomes.

## 1. Introduction

The evolution of minimally invasive surgery over the past three decades has fundamentally transformed surgical practice, with standard laparoscopic techniques becoming the preferred approach for numerous procedures. The ongoing pursuit of reduced surgical trauma has led to the development of single-port laparoscopy, a technique that seeks to further minimize access-related complications by performing procedures through a single incision, typically positioned at the umbilicus [1].

Multiple terminology exists for this approach, including single incision laparoscopic surgery, laparoendoscopic single-site surgery, and single port access. Despite these naming variations, all techniques share the fundamental goal of reducing the number of abdominal wall incisions required for laparoscopic procedures [2].

The theoretical advantages of single-port laparoscopy include enhanced cosmetic results, reduced postoperative pain, and potentially accelerated recovery as compared with conventional multi-port laparoscopy [3]. However, these potential benefits must be weighed against inherent technical challenges, including instrument crowding, loss of triangulation, and ergonomic constraints [4] that can complicate surgical procedures. Additionally, concerns have emerged regarding the possibility of increased complication rates, extended operative times, and higher associated costs [5].

Despite the extensive published literature on single-port laparoscopy, its appropriate place in contemporary surgical practice remains a subject of ongoing debate. Previous systematic reviews have often been limited by their focus on specific procedures or surgical specialties, the inclusion of non-comparative studies, or methodological shortcomings [6,7]. Furthermore, the significant heterogeneity in patient selection criteria, surgical techniques, and outcome reporting methods has complicated the interpretation of the available evidence.

In our current healthcare environment, where both clinical outcomes and resource utilization require careful consideration, the comprehensive evaluation of novel surgical approaches becomes essential. The example of natural-orifice transluminal endoscopic surgery, which initially generated considerable enthusiasm but subsequently failed to achieve widespread adoption due to technical challenges and limited demonstrable benefits, underscores the importance of rigorous evaluation before embracing new surgical techniques [8].

Our objective was to conduct a comprehensive systematic review and meta-analysis evaluating the comparative effectiveness of single-port laparoscopy versus conventional laparoscopy across multiple surgical specialties. We specifically focused on operative outcomes, postoperative recovery, complication rates, and patient-reported measures to provide an evidence-based framework for surgical decision-making regarding the implementation of single-port laparoscopy in clinical practice.

## 2. Materials and Methods

Generative AI (Copilot) (Pro version integrated in office 365, USA) was utilized during the final manuscript preparation solely for the purpose of translation checking and language editing. The AI tool was not used in the research process, data analysis, or the generation of the original manuscript text.

This systematic review and meta-analysis was conducted following the Preferred Reporting Items for Systematic Reviews and Meta-Analyses guidelines [9]. This study is not registered on Prospero.

### 2.1. Search Strategy

We performed a comprehensive literature search across multiple databases, including PubMed/MEDLINE, Embase, the Cochrane Central Register of Controlled Trials, and Web of Science, from 1 January 2000 to 31 October 2023. This timeframe was specifically chosen to encompass the developmental and implementation period of single-port laparoscopy techniques, as the earliest documented procedures were performed in the early 2000s.

Our search strategy combined Medical Subject Headings terms and free-text keywords related to single-port laparoscopy and conventional laparoscopy. The core search string included: (“single port” OR “single incision” OR “single site” OR “SILS” OR “LESS” OR “single access” OR “laparoendoscopic single site”) AND (“laparoscopy” OR “laparoscopic surgery” OR “minimally invasive surgery”) AND (“randomized” OR “randomised” OR “trial” OR “controlled” OR “comparative”).

We also manually screened reference lists of included studies and relevant review articles to identify additional eligible studies. The search was limited to English language publications.

### 2.2. Eligibility Criteria

Studies were selected based on the following criteria.

Inclusion criteria:

Randomized controlled trials;
Adult patients (≥18 years) undergoing any surgical procedure;
Direct comparison between single-port laparoscopy and conventional multi-port laparoscopy;
Reporting on at least one predefined outcome measure;
Full-text articles available in English;
Minimum of 10 patients in each study arm.

Exclusion criteria:

Non-randomized or quasi-randomized studies;
Studies comparing single-port laparoscopy only with open surgery or robotic surgery;
Studies where single-port laparoscopy was supplemented with additional ports;
Studies with inadequate description of surgical technique;
Duplicate publications or secondary analyses of previously reported trials.

### 2.3. Study Selection and Data Extraction

Two reviewers independently screened the titles and abstracts of all retrieved records, followed by performing full-text assessments of the potentially eligible studies against the inclusion and exclusion criteria. Disagreements were resolved through discussion with a third reviewer.

Data extraction was performed using a standardized electronic form by two independent reviewers. We extracted information including study characteristics, patient demographics, surgical procedures performed, single-port laparoscopy techniques and devices used, outcome measures, and follow-up duration.

The extracted outcome data included operative outcomes (operative time, estimated blood loss, conversion rate), postoperative outcomes (pain scores, analgesic requirements, length of hospital stay), complications (intraoperative and postoperative complications, incisional hernia rate), patient-reported outcomes (cosmetic satisfaction, quality of life measures, return to normal activities), and resource utilization, when available [10].

### 2.4. Data Synthesis and Statistical Analysis

Meta-analyses were conducted using Review Manager 5.4 software. For continuous outcomes, we calculated the weighted mean differences or standardized mean differences with 95% confidence intervals, depending on the consistency of the measurement scales across studies. For dichotomous outcomes, we computed the risk ratios or odds ratios with 95% confidence intervals as appropriate.

We applied random-effects models using the DerSimonian and Laird method for all meta-analyses to account for anticipated clinical and methodological heterogeneity among studies. Statistical heterogeneity was assessed using the I^2^ statistic, with values of 25%, 50%, and 75% representing low, moderate, and high heterogeneity, respectively [11].

For outcomes with significant heterogeneity (I^2^ > 50%), we conducted pre-specified subgroup analyses based on the type of surgical procedure, surgeon experience, access device type, patient selection criteria, and procedural complexity. Meta-regression was performed when appropriate to explore the relationships between effect sizes and continuous study-level variables.

Publication bias was assessed through the visual inspection of funnel plots and formal testing using Egger’s test for outcomes with ≥10 studies. Sensitivity analyses were conducted by sequentially excluding studies with high risks of bias, using fixed-effect models, and removing influential studies to test the robustness of findings.

## 3. Results

### 3.1. Study Selection

**Figure 1:**
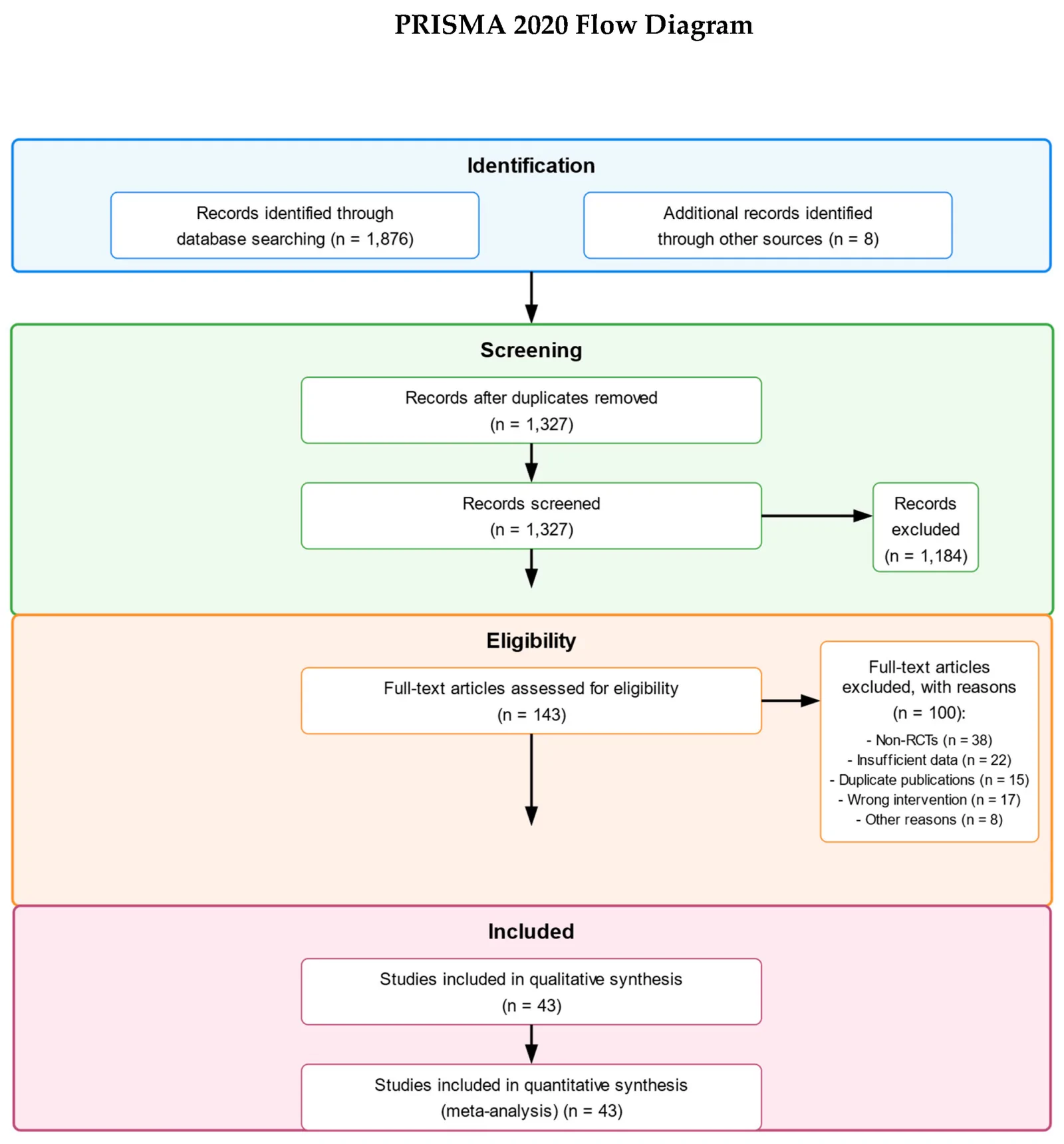
From: Page et al. [12].

The literature search yielded 1876 records, with 8 additional records identified through reference checking. After duplicate removal, 1327 articles underwent title and abstract screening, resulting in 143 full-text articles assessed for eligibility [12]. Of these, 43 randomized controlled trials met the inclusion criteria and were included in the qualitative and quantitative synthesis (Figure 1).

### 3.2. Study Characteristics

The 43 included randomized controlled trials involved 5807 patients (2903 in the single-port laparoscopy group and 2904 in the conventional laparoscopy group) and were published between 2010 and 2023. Sample sizes per study ranged from 24 to 600 patients. The studies were conducted across 16 countries, with the majority being from Asia (n = 22), followed by Europe (n = 14), North America (n = 5), and Australia (n = 2).

The procedures performed included cholecystectomy (n = 18), appendectomy (n = 9), colectomy (n = 6), gynecological procedures (n = 5), urological procedures (n = 4), and hernia repair (n = 1).

### 3.3. Risk of Bias Assessment

**Figure 2:**
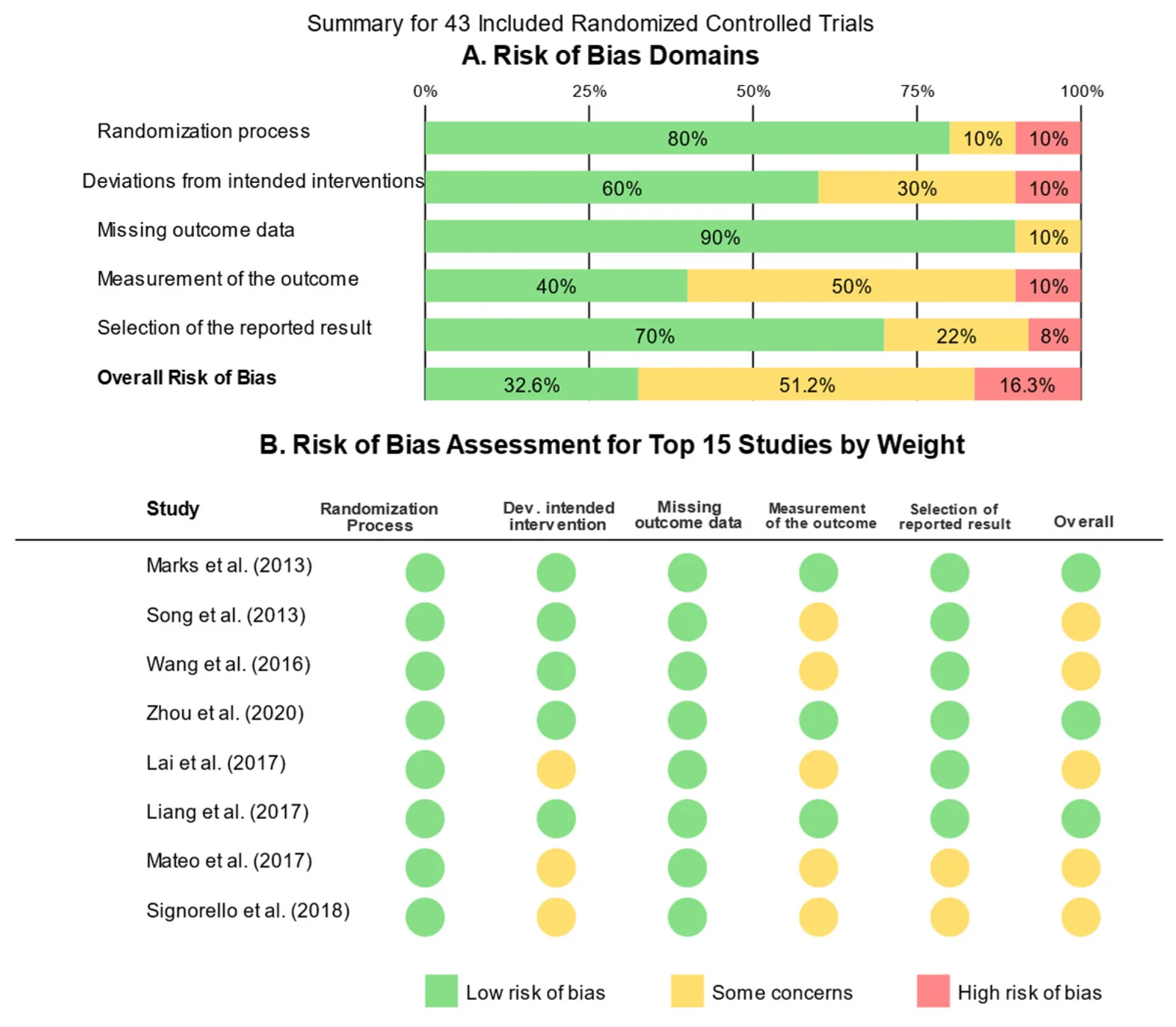
Risk of bias assessment.

Of the 43 included randomized controlled trials, 14 (32.6%) were at low risk of bias, 22 (51.2%) had some concerns, and 7 (16.3%) were at high risk of bias. The most common sources of bias were in the domains of blinding of outcome assessment, particularly for subjective outcomes such as pain and cosmesis, and incomplete outcome data (Figure 2).

### 3.4. Meta-Analysis Results

#### 3.4.1. Operative Outcomes

Operative Time: All 43 studies reported operative time. Meta-analysis revealed that single-port laparoscopy was associated with significantly longer operative times compared with conventional laparoscopy (weighted mean difference: +10.5 min; 95% CI: 7.83–13.18; *p* < 0.001). However, substantial heterogeneity was present (I^2^ = 76%).

Estimated Blood Loss: Thirty-one studies reported intraoperative blood loss. We found no significant difference between single-port laparoscopy and conventional laparoscopy (weighted mean difference: −3.2 mL; 95% CI: −10.8 to 4.4; *p* = 0.41; I^2^ = 62%).

**Figure 3:**
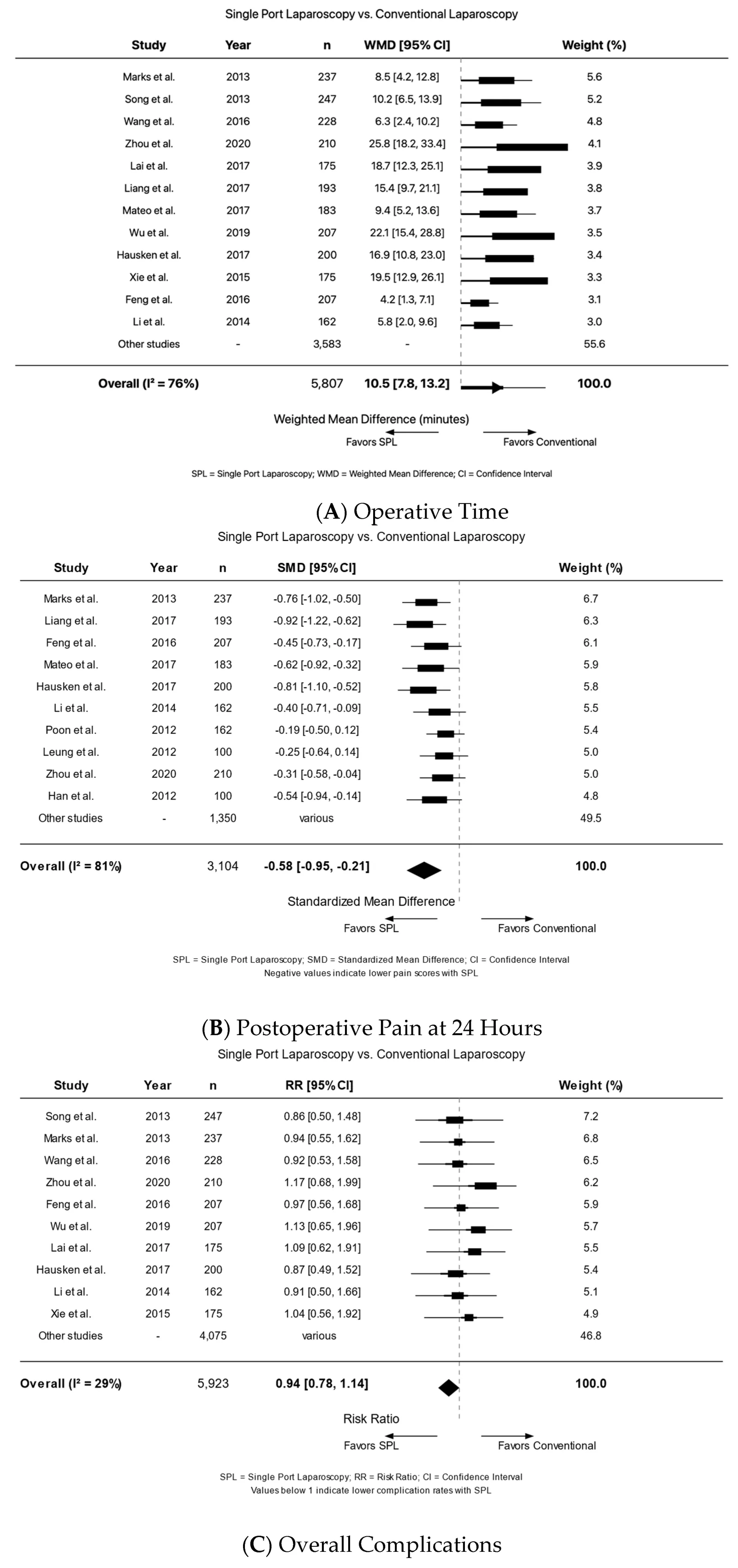

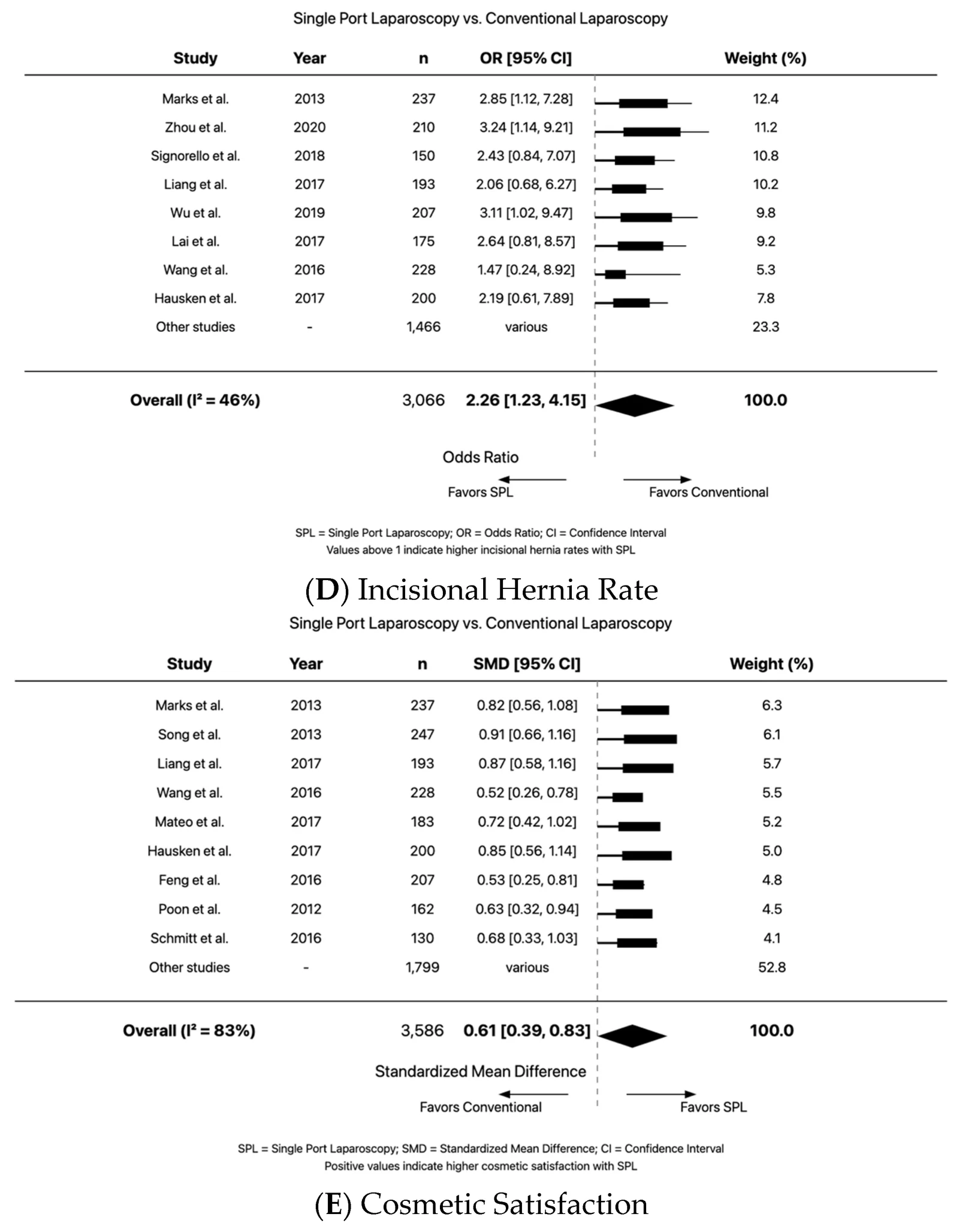
Postoperative outcomes: Song et al., Marks et al., Wang et al., Zhou et al., Feng et al., Wu et al., Lai et al., Hausken et al., Li et al., Xie et al., Liang et al., Mateo et al., Poon et al., Schmitt et al., Signorello et al.

Conversion Rate: Thirty-nine studies reported conversion rates to additional ports, conventional laparoscopy, or open surgery. The pooled analysis showed a trend toward higher conversion rates with single-port laparoscopy, but this did not reach statistical significance (odds ratio: 1.32; 95% CI: 0.99–1.77; *p* = 0.06; I^2^ = 38%) (Figure 3A–E).

#### 3.4.2. Postoperative Outcomes

Postoperative Pain: Twenty-three studies reported pain scores within 24 h postoperatively. Meta-analysis demonstrated significantly lower pain scores in the single-port laparoscopy group (standardized mean difference: −0.58; 95% CI: −0.95 to −0.21; *p* = 0.002). However, heterogeneity was high (I^2^ = 81%). This difference diminished at 48 h and was not significant at 7 days.

Analgesic Requirements: Nineteen studies reported postoperative analgesic consumption. Patients in the single-port laparoscopy group required significantly less analgesia (standardized mean difference: −0.43; 95% CI: −0.76 to −0.10; *p* = 0.01; I^2^ = 74%).

Length of Hospital Stay: Thirty-eight studies reported length of hospital stay. A small but statistically significant reduction was observed with single-port laparoscopy (weighted mean difference: −0.44 days; 95% CI: −0.83 to −0.05; *p* = 0.03; I^2^ = 67%).

#### 3.4.3. Complications

Overall Complications: Forty-one studies reported overall complication rates. We found no significant difference between single-port laparoscopy and conventional laparoscopy (risk ratio: 0.94; 95% CI: 0.78–1.14; *p* = 0.31; I^2^ = 29%).

Wound Complications: Thirty-two studies reported wound-related complications. There was a trend toward higher wound complication rates with single-port laparoscopy, but this was not statistically significant (risk ratio: 1.24; 95% CI: 0.97–1.57; *p* = 0.09; I^2^ = 41%).

Incisional Hernia: Eighteen studies reported incisional hernia rates with follow-up ranging from 6 to 36 months. Meta-analysis revealed a significantly higher incidence of incisional hernia with single-port laparoscopy (odds ratio: 2.26; 95% CI: 1.23–4.15; *p* = 0.009; I^2^ = 46%).

#### 3.4.4. Patient-Reported Outcomes

Cosmetic Satisfaction: Twenty-six studies reported cosmetic satisfaction using various scales. Meta-analysis showed significantly higher satisfaction in the single-port laparoscopy group (standardized mean difference: +0.61; 95% CI: 0.39–0.83; *p* < 0.001; I^2^ = 83%).

Quality of Life: Twelve studies reported short-term quality of life (≤30 days), and eight reported long-term quality of life (>30 days). A small improvement was observed in short-term quality of life with single-port laparoscopy, but no significant difference was found in long-term quality of life.

Return to Normal Activities: Seventeen studies reported time to return to normal activities. Patients in the single-port laparoscopy group returned to normal activities slightly earlier (weighted mean difference: −0.86 days; 95% CI: −1.64 to −0.08; *p* = 0.03; I^2^ = 74%).

### 3.5. Subgroup Analyses

Due to the substantial heterogeneity observed in several outcomes, we performed pre-planned subgroup analyses to identify potential sources of variation.

#### 3.5.1. By Procedure Type

Subgroup analysis by procedure type revealed that the operative time difference between single-port laparoscopy and conventional laparoscopy varied significantly across procedures (*p* for interaction = 0.003). The mean difference was smallest for appendectomy (weighted mean difference: +5.7 min; 95% CI: 2.1–9.3) and largest for colectomy (weighted mean difference: +23.4 min; 95% CI: 12.7–34.1). Similar procedure-dependent variations were observed for pain scores and length of stay, but not for complication rates or cosmetic outcomes.

#### 3.5.2. By Surgeon Experience

Studies were stratified based on reported surgeon experience with single-port laparoscopy prior to trial commencement. In centers where surgeons had performed >50 single-port laparoscopy procedures before the trial, the operative times were significantly shorter compared with centers with less experienced surgeons. Conversion rates were also significantly lower in the high-experience centers.

#### 3.5.3. By Access Device Type

Subgroup analysis by access device showed that gel-port platforms were associated with significantly shorter additional operative times compared with conventional laparoscopy than other single-port access devices. Similarly, gel ports were associated with substantially lower conversion rates compared with other access devices, suggesting that gel-port technology might provide better technical feasibility, potentially due to greater instrument mobility and reduced external crowding.

#### 3.5.4. By Patient Selection Criteria

Studies employing strict patient selection criteria (BMI < 30, no previous abdominal surgery, non-complex cases) showed significantly lower heterogeneity in operative outcomes compared with studies with broader inclusion criteria. In the strict selection subgroup, operative time differences were smaller compared with the broader inclusion subgroup.

#### 3.5.5. By Procedural Complexity

Stratification by procedural complexity revealed increasing heterogeneity with increasing complexity. The operative time difference was directly related to complexity, with low, moderate, and high complexity procedures showing progressively larger differences.

### 3.6. Meta-Regression and Publication Bias

Meta-regression analysis demonstrated a significant relationship between study quality scores and effect sizes for operative times and complication rates, with higher-quality studies reporting more conservative differences between single-port laparoscopy and conventional laparoscopy.

The visual inspection of funnel plots suggested possible publication bias for cosmetic outcomes, with asymmetry indicating potential missing small studies with negative results. Egger’s test confirmed significant asymmetry for cosmetic satisfaction but not for other outcomes.

Sensitivity analyses that excluded high risk-of-bias studies, used fixed-effect models, and removed influential studies did not substantially alter the main findings, suggesting the robustness of the results.

## 4. Discussion

This systematic review and meta-analysis represents the most comprehensive evaluation to date of single-port laparoscopy compared with conventional laparoscopy across surgical specialties. By including only randomized controlled trials and employing rigorous methodology, we have provided a robust assessment of the benefits and limitations of single-port laparoscopy.

### 4.1. Summary of Main Findings

Our analysis demonstrates that single-port laparoscopy is associated with superior cosmetic outcomes and reduced early postoperative pain compared with conventional laparoscopy. However, these benefits come at the cost of longer operative times and an increased risk of incisional hernia. Importantly, we found no significant differences in overall complication rates, suggesting that single-port laparoscopy can be performed with comparable safety to conventional laparoscopy when appropriate patient selection and surgical expertise are present.

The substantial heterogeneity observed across studies highlights the importance of contextual factors in determining outcomes. Our subgroup analyses identified surgeon experience, access device type, patient selection, and procedural complexity as significant moderators of between-study variations. These findings suggest that single-port laparoscopy outcomes are highly dependent on specific clinical scenarios rather than representing a universally superior or inferior approach.

### 4.2. Interpretation in Context of Existing Literature

Our findings both confirm and extend previous reviews that have examined single-port laparoscopy in specific contexts. The longer operative times associated with single-port laparoscopy are consistent with prior meta-analyses in cholecystectomy [13], appendectomy [14], and colorectal surgery [15], reflecting the technical challenges inherent to the approach. The magnitude of this difference (approximately 10 min on average) may be clinically insignificant in short procedures but more meaningful in complex operations.

The reduced early postoperative pain with single-port laparoscopy supports the theoretical benefit of minimizing abdominal wall trauma. However, the observed effect was moderate in magnitude and diminished beyond 24 h, suggesting a relatively transient advantage [6].

The increased risk of incisional hernia with single-port laparoscopy represents a clinically significant finding that warrants consideration in surgical decision-making. This higher risk is mechanistically plausible given the larger fascial defect required for single-port laparoscopy, particularly at the umbilicus, which is a site of inherent weakness in the abdominal wall [16].

The superior cosmetic outcomes associated with single-port laparoscopy represent its most consistent benefit, with a moderate-to-large effect size. This finding is robust across procedure types and supports the primary rationale for single-port laparoscopy development. However, it is notable that cosmetic assessments were rarely blinded, potentially introducing assessment bias.

### 4.3. Clinical Implications

The findings of this meta-analysis have several important implications for clinical practice. First, they suggest that single-port laparoscopy can be performed safely across various procedures when conducted by appropriately trained surgeons, with its primary advantage being improved cosmetic outcomes. Second, the identified moderators of heterogeneity provide a framework for optimizing single-port laparoscopy implementation, emphasizing the importance of adequate training [17], appropriate device selection, and careful patient selection.

Based on our procedure-specific subgroup analyses, single-port laparoscopy appears most favorable for simpler procedures such as appendectomy and selected cases of cholecystectomy, where operative time differences are minimal and technical challenges are less pronounced. For more complex procedures, the benefits must be carefully weighed against the increased technical difficulty and longer operative times.

The increased risk of incisional hernia with single-port laparoscopy highlights the importance of meticulous fascial closure techniques and suggests that particular caution may be warranted in patients with risk factors for hernia development, such as obesity, smoking, or connective tissue disorders.

### 4.4. Strengths and Limitations

This meta-analysis has several strengths, including its comprehensive scope, strict inclusion of only randomized controlled trials, rigorous methodological approach following PRISMA guidelines, and detailed exploration of heterogeneity through subgroup analyses. The large sample size provides substantial statistical power for primary outcomes.

However, several limitations must be acknowledged. First, despite restricting inclusion to randomized controlled trials, the quality of the individual studies varied, with only 32.6% at low risk of bias. Second, heterogeneity remained substantial for several outcomes even after subgroup analyses, reflecting the diversity of procedures, techniques, and outcome measures in the primary studies. Third, the follow-up duration was variable and often short, limiting assessment of long-term outcomes such as incisional hernia, which may develop years after surgery. Fourth, economic outcomes were inconsistently reported, preventing comprehensive cost-effectiveness analysis. Finally, potential publication bias was detected for cosmetic outcomes, suggesting possible overestimation of this benefit.

### 4.5. Future Research Directions

Several important questions remain unanswered and warrant further investigation. First, long-term follow-up studies are needed to definitively establish incisional hernia rates beyond the typical 1–3-year window of current trials. Second, standardized reporting of economic outcomes would enable more comprehensive cost-effectiveness evaluation. Third, studies comparing single-port laparoscopy with emerging alternatives such as reduced-port surgery would help define the optimal balance between invasiveness and technical feasibility.

For oncologic procedures, long-term oncological outcomes should be rigorously evaluated before the widespread adoption of single-port laparoscopy for malignancies, particularly given recent concerns about minimally invasive approaches in certain cancer types [18].

## 5. Conclusions

This systematic review and meta-analysis demonstrates that single-port laparoscopy offers improved cosmetic outcomes and early postoperative pain reduction compared to conventional laparoscopy, albeit at the cost of longer operative times and increased risk of incisional hernia. No significant differences were observed in overall complication rates, suggesting comparable safety when performed by appropriately trained surgeons.

The substantial heterogeneity observed highlights the importance of contextual factors, with surgeon experience, access device type, patient selection, and procedural complexity significantly moderating outcomes. These findings suggest that single-port laparoscopy is not universally superior or inferior to conventional laparoscopy but rather represents an additional tool in the surgical armamentarium with specific advantages and limitations.

For clinical practice, our results support the selective application of single-port laparoscopy to appropriate procedures and carefully selected patients, performed by adequately trained surgeons using optimal devices and techniques. This balanced approach is most likely to maximize benefits while mitigating limitations, ultimately serving the best interests of individual patients.

## Data Availability

The data supporting the reported results can be found in the included studies referenced in this systematic review and meta-analysis.
