# Correction: Mussa et al. Single-Port Laparoscopy Compared with Conventional Laparoscopic Surgery: A Systematic Review and Meta-Analysis. *J. Clin. Med.* 2025, *14*, 4915

**DOI:** 10.3390/jcm15218203

**Published:** 2026-10-22

**Authors:** Baudolino Mussa, Barbara Defrancisco, Ludovico Campi, Mario Morino

**Affiliations:** 1Surgical Science Department, https://ror.org/048tbm396University of Turin, 10124 Turin, Italy; ludovico.campi@edu.unito.it (L.C.); mario.morino@unito.it (M.M.); 2Alchemica Torino, Via San Marino 25, 10136 Turin, Italy; barbyd77@gmail.com

## Table Correction

There was an omission in the original publication [1]. The manuscript did not include a disclosure of the use of generative artificial intelligence tools during manuscript preparation, as required by MDPI editorial policy.

A correction has been made to Section 2 (Materials and Methods), inserted as a new paragraph at the beginning of the section:

“Generative AI (Copilot) (Pro version integrated in office 365, USA) was utilized during the final manuscript preparation solely for the purpose of translation checking and language editing. The AI tool was not used in the research process, data analysis, or the generation of the original manuscript text.”

## References

In the original publication [1], the authors used an AI assistant, Copilot (Pro version integrated in office 365 USA), to help check the translation and perform editorial adaptations, during which errors were unintentionally introduced: the tool incorrectly modified several citations, in some cases creating references that do not exist. References [1–5,7–9,13–17] contained formatting inconsistencies, including mixed citation styles, inconsistent punctuation, and non-standard abbreviations. References [1–5,7–9,13–17] are thus replaced. All references have been corrected to comply with MDPI reference formatting guidelines. The corrected reference list is provided below. The order of some references has been adjusted accordingly.

Corrected References


Blanco, F.C.; Kane, T.D. Single-port laparoscopic surgery in children: Concept and controversies of the new technique. *Minim. Invasive Surg.*
**2012**, *2012*, 232347. https://doi.org/10.1155/2012/232347.Autorino, R.; Kim, F.J. Urologic Laparoendoscopic Single-Site Surgery (LESS): Current status. *Urologia*
**2011**, *78*, 32–41. https://doi.org/10.5301/ru.2011.6448.Pereira, C.; Gururaj, S. A Systematic Review and Meta-Analysis of Single-Incision Laparoscopic Cholecystectomy Versus Conventional Four-Port Laparoscopic Cholecystectomy. *Cureus*
**2022**, *14*, e32524. https://doi.org/10.7759/cureus.32524.Kirshtein, B.; Haas, E.M. Single port laparoscopic surgery: Concept and controversies of new technique. *Minim. Invasive Surg.*
**2012**, *2012*, 456541. https://doi.org/10.1155/2012/456541.Lyu, Y.; Cheng, Y.; Wang, B.; Zhao, S.; Chen, L. Single-incision versus conventional multiport laparoscopic cholecystectomy: A current meta-analysis of randomized controlled trials. *Surg. Endosc.*
**2020**, *34*, 4315–4329. https://doi.org/10.1007/s00464-019-07198-8.Ahmed, K.; Wang, T.T.; Patel, V.M.; Nagpal, K.; Clark, J.; Ali, M.; Deeba, S.; Ashrafian, H.; Darzi, A.; Athanasiou, T.; et al. The role of single-incision laparoscopic surgery in abdominal and pelvic surgery: A systematic review. *Surg. Endosc.*
**2011**, *25*, 378–396. https://doi.org/10.1007/s00464-010-1208-6.Rudiman, R.; Hanafi, R.V.; Almawijaya, A. Single-site robotic cholecystectomy versus single-incision laparoscopic cholecystectomy: A systematic review and meta-analysis. *Ann. Gastroenterol. Surg.*
**2023**, *7*, 709–718. https://doi.org/10.1002/ags3.12688.Autorino, R.; Yakoubi, R.; White, W.M.; Gettman, M.; De Sio, M.; Quattrone, C.; Di Palma, C.; Izzo, A.; Correia-Pinto, J.; Kaouk, J.H.; et al. Natural orifice transluminal endoscopic surgery (NOTES): Where are we going? A bibliometric assessment. *BJU Int.*
**2013**, *111*, 11–16. https://doi.org/10.1111/j.1464-410X.2012.11494.x.Moher, D.; Liberati, A.; Tetzlaff, J.; Altman, D.G.; PRISMA Group. Preferred reporting items for systematic reviews and meta-analyses: The PRISMA statement. *PLoS Med.*
**2009**, *6*, e1000097. https://doi.org/10.1371/journal.pmed.1000097.Sterne, J.A.C.; Savović, J.; Page, M.J.; Elbers, R.G.; Blencowe, N.S.; Boutron, I.; Cates, C.J.; Cheng, H.Y.; Corbett, M.S.; Eldridge, S.M.; et al. RoB 2: A revised tool for assessing risk of bias in randomised trials. *BMJ*
**2019**, *366*, l4898. https://doi.org/10.1136/bmj.l4898.Higgins, J.P.T.; Thompson, S.G.; Deeks, J.J.; Altman, D.G. Measuring inconsistency in meta-analyses. *BMJ*
**2003**, *327*, 557–560. https://doi.org/10.1136/bmj.327.7414.557.Page, M.J.; McKenzie, J.E.; Bossuyt, P.M.; Boutron, I.; Hoffmann, T.C.; Mulrow, C.D.; Shamseer, L.; Tetzlaff, J.M.; Akl, E.A.; Brennan, S.E.; et al. The PRISMA 2020 statement: An updated guideline for reporting systematic reviews. *BMJ*
**2021**, *372*, n71. https://doi.org/10.1136/bmj.n71.Pisanu, A.; Reccia, I.; Porceddu, G.; Uccheddu, A. Meta-analysis of prospective randomized studies comparing single-incision laparoscopic cholecystectomy (SILC) and conventional multiport laparoscopic cholecystectomy (CMLC). *J. Gastrointest. Surg.*
**2012**, *16*, 1790–1801. https://doi.org/10.1007/s11605-012-1956-9.Li, P.; Chen, Z.H.; Li, Q.G.; Qiao, T.; Tian, Y.Y.; Wang, D.R. Safety and efficacy of single-incision laparoscopic surgery for appendectomies: A meta-analysis. *World J. Gastroenterol.*
**2013**, *19*, 4072–4082. https://doi.org/10.3748/wjg.v19.i25.4072.Yuan, Y.; Jian, J.; Jing, H.; Yan, R.; You, F.; Fu, X.; Du, L.; Li, W. Single-Incision vs. Conventional Laparoscopic Surgery for Colorectal Cancer: An Update of a Systematic Review and Meta-Analysis. *Front. Surg.*
**2021**, *8*, 704986. https://doi.org/10.3389/fsurg.2021.704986.Connell, M.B.; Selvam, R.; Patel, S.V. Incidence of incisional hernias following single-incision versus traditional laparoscopic surgery: A meta-analysis. *Hernia*
**2019**, *23*, 91–100. https://doi.org/10.1007/s10029-018-1853-6.Ma, J.; Yang, J.; Cheng, S.; Jin, Y.; Zhang, N.; Wang, C.; Wang, Y. The Learning Curve of Laparoendoscopic Single-Site Surgery in Benign Gynecological Diseases. *J. Investig. Surg.*
**2022**, *35*, 363–370. https://doi.org/10.1080/08941939.2020.1867673.Ramirez, P.T.; Frumovitz, M.; Pareja, R.; Lopez, A.; Vieira, M.; Ribeiro, M.; Buda, A.; Yan, X.; Shuzhong, Y.; Chetty, N.; et al. Minimally invasive versus abdominal radical hysterectomy for cervical cancer. *N. Engl. J. Med.*
**2018**, *379*, 1895–1904. https://doi.org/10.1056/NEJMoa1806395. PMID: 30380365.


The authors state that the scientific conclusions are unaffected. This correction was approved by the Academic Editor. The original publication has also been updated.
